# *In Vitro* and *In Vivo* Enhancement of Adipogenesis by Italian Ryegrass (*Lolium multiflorum*) in 3T3-L1 Cells and Mice

**DOI:** 10.1371/journal.pone.0085297

**Published:** 2014-01-13

**Authors:** Mariadhas Valan Arasu, Soundharrajan Ilavenil, Da Hye Kim, Sang Gun Roh, Jeong-Chae Lee, Ki Choon Choi

**Affiliations:** 1 Grassland and Forage Division, National Institute of Animal Science, RDA, Cheonan, South Korea; 2 The United Graduate School of Agricultural Sciences, Tottori University, Tottori-Shi, Japan; 3 Laboratory of Animal Physiology, Graduate School of Agricultural Science, Tohoku University, Sendai, Japan; 4 Research Center of Bioactive Materials, Institute of Oral Biosciences and School of Dentistry, Chonbuk National University, Jeonju, South Korea; Virgen Macarena University Hospital, School of Medicine, University of Seville, Spain

## Abstract

Adipogenesis is very much important in improving the quality of meat in animals. The aim of the present study was to investigate the *in vitro* and *in vivo* adipogenesis regulation properties of *Lolium multiflorum* on 3T3-L1 pre-adipocytes and mice. Chemical composition of petroleum ether extract of *L. multiflorum* (PET-LM) confirmed the presence of fatty acids, such as α-linolenic acid, docosahexaenoic acid, oleic acid, docosatetraenoic acid, and caprylic acid, as the major compounds. PET-LM treatment increased viability, lipid accumulation, lipolysis, cell cycle progression, and DNA synthesis in the cells. PET-LM treatment also augmented peroxysome proliferator activated receptor (PPAR)-γ2, CCAAT/enhancer binding protein-α, adiponectin, adipocyte binding protein, glucose transporter-4, fatty acid synthase, and sterol regulatory element binding protein-1 expression at mRNA and protein levels in differentiated adipocytes. In addition, mice administered with 200 mg/kg body weight PET-LM for 8 weeks showed greater body weight than control mice. These findings suggest that PET-LM facilitates adipogenesis by stimulating PPARγ-mediated signaling cascades in adipocytes which could be useful for quality meat development in animals.

## Introduction

Farm animal cultivars pay much attention in the development of high quality meat for attracting the customers in the market. The diet is a key factor in modulating the quality and quantity of meat on cattle, goats, and sheep [1]–[3]. However, feed costs represent the largest single variable cost in meat production in Korea. The meat quality trait has a unique process which controlled by the multi-factorial events. The diet containing higher lipid level induces fat accumulation in animals. Generally, lipid accumulation and adipose tissue development are continuous processes and depend on the genetic, hormonal, and type of dietary factors.

Adipogenesis is an important process which enhances the lipid accumulation in muscles and other tissues. This process is very much essential for quality meat development in animals because Intramuscular fat (marbling) is an important criterion for quality meat development in animals because marbling is an associated with the number of the adipocytes or fat cells. Adipocyte proliferation and differentiation are usually alternative and mutually exclusive pathways [4], [5]. Many transcriptional factors such as peroxysome proliferator activated receptor (PPAR)-γ, CCAAT/enhancer binding protein-β (C/EBP-β), C/EBP-δ, C/EBP-α, Kriuppel like factor, and sterol regulatory element binding protein-1 (SREBP-1) are mainly involved in promoting proliferation and differentiation of adipocytes [6]. During adipogenic differentiation, C/EBP-β and C/EBP-δ are induced immediately by glucorticoids and insulin, and then they activate PPARγ and C/EBP-α. The final stage of adipocyte differentiation requires the expression of genes which are involved in the adipocyte phenotype and functional maintenance via lipid metabolic enzymes. A number of genes were involved in the lipid metabolism. Fatty acid binding protein-4 (aP2) is a key factor for intracellular fatty acid transport and lipid metabolism. The aP2 is regulated by PPARγ and C/EBP-α and is a key marker in the terminal steps of adipocytes differentiation [7], [8]. PPARγ modulates the insulin-signaling pathway through up-regulation of many factors involved in the signaling cascade from glucose transporter (GLUT)-4 which enhances the glucose uptake levels by the cells. Adiponectin, called adipo-Q or adipocyte complement-related protein, is specifically expressed in adipose tissue during differentiation [9]. Adiponectin enhances the sensitivity in muscle and liver and also increases free fatty acid oxidation in several tissues including muscle fiber [10], [11].

Italian Ryegrass (IRG) is being cultivated in South Korea to produce silage for domestic animals. Most of the farmers in South Korea have cultivated IRG as a feed for ruminants, because of its higher nutritive value and fast growing nature even under a cold climate. The nutritional quality of IRG is varied with other varieties. Farmers purchases many types of forage products from companies to use as a feed for ruminants, but the cost in using commercial feed is relatively expensive than IRG-based silage is used. We preliminarily investigated the effects of IRG silage/concentrate (TRT) on the growth performance, feed intake, slaughter characteristics, and its meat quantity and quality characteristics of *M. longissimusdorsi* (Hanwoo steer). The body weight, higher back-fat thickness, and rib-eye area increased more highly in TRT fed animals than the control animal (data not shown). This effect of IRG-based feed is considered to be associated with the high protein and fatty acid contents in IRG. Therefore, we investigated the chemical composition of petroleum ether extract of *L. multiflorum* (PET-LM) and explored its effects on adipocyte proliferation and differentiation using a 3T3-L1 cell line. We also examined the possible mechanisms by which PET-LM positively regulates adipogenesis and thus increases body weight of animal.

## Materials and Methods

### Chemicals

Mouse 3T3-L1 pre-adipocyte cell line was purchased from the American Type Culture Collection (Rockwille, MD, USA). Fetal bovine serum (FBS) and Dulbecco modified Eagle medium (DMEM) were purchased from Gibco-BRL (Gaithersburg, MD, USA). Kits for mRNA extraction and RT-PCR were obtained from Invitrogen (Carlsbad, CA, USA). Unless specified otherwise, other chemicals and laboratory wares procured from Sigma Chemical Co. (St. Louis, MO, USA) and SPL Life Sciences (Pochun, South Korea).

### Plant Material and Extraction

*L. multiflorum* was collected from National Institute of Animal Science (NIAS), South Korea, at September in 2011. The plant was identified and authenticated by a plant taxonomist at NIAS. The *L. multiflorum* was harvested at flowering stage, ensiled at 40 days after cultivation, and then air-dried under shadow before grinding to obtain a coarse powder. The powder (5 kg) was soaked in 10 L of petroleum ether and incubated at room temperature for 72 h by intermittently mixing in an orbital shaker. The mixture was filtered using Whatman filter paper and the filtrates were concentrated using rotary evaporator at 40°C until organic solvent was completely removed. The resulting powder, named PET-LM, was stored under 4°C before use in subsequent experiments.

### LC-MS/MS Analysis of PET-LM

An API 4000 Q TRAP tandem mass spectrometer (Applied Biosystems, Foster City, CA, USA), equipped with an Agilent 1200 series HPLC system (Agilent Technologies, Palo Alto, CA, USA) and an electro spray ionization tandem mass spectrometry (Micromass Quattromicro API, Waters Corporation, Milford, MA, USA), was used to identify the fatty acid profiles in PET-LM. This study used ACQUITY BEH C18 column (1.7 µm) and 20 mM ammonium acetate and acetonitrile containing 0.1% formic acid as solvent A and B, respectively. The analytical conditions of mass spectrometry were: Scan range, PDA: 200∼500 nm and MS: *m*/*z* 100∼1000; capillary voltage, 3.0 kV; cone voltage, 20 V and 30 V; source temperature, 120°C; desolvation temp, 300°C.

### Cell Culture and Treatment

Adipocyte differentiation induction was carried out by the method of Choi *et al*. [12] with a slight modification. Briefly, 3T3-L1 pre-adipocytes were seeded in 6 well culture plats at a density of 3×10^4^ cells/well. Cells were incubated at 37°C in an incubator containing 5% CO_2_ and culture medium was replenished every 48 h. When the cells reached to >95% confluence, growth medium was replaced to differentiation medium (DMEM containing 10% FBS, 0.5 mM 3-isobutyl-1-methylxanthine, 1 μM dexamethasone, and 1.7 μM insulin). After 48 h, the medium was switched to post differentiation medium and then further incubated for various times (0–10 days) in the presence of various PET-LM concentrations (0–100 μg/ml). The media was changed newly to the same media containing PET-LM every two days for the incubation periods. Subsequently, these cells were adjusted to the assays for analyzing differentiation, lipolysis, and adipogenic factor expression at mRNA and protein levels.

### Oil Red O Staining

PET-LM-treated cells were fixed with 10% formalin for 1 h and then washed with 60% isopropanol. Oil Red O solution (3 ml/well) was added into the plates and the cells were incubated at room temperature for 10 min. Cells were washed three times with distilled water and photographed using an inverted microscope (CKX41, Olympus Corporation, Tokyo, Japan). In addition, Oil Red O was extracted from the cells with 100% isopropanol and the absorbance of the dye was measured at 490 nm using a microplate reader (Packard Instrument Co., Downers Grove, IL, USA).

### Measurements of Glycerol Production

Lipolysis was determined by measuring the accumulation of glycerol using an Adipolysis Assay Kit (CHEMICON® International Inc., Temecula, CA, USA). Briefly, the differentiated 3T3-L1 cells in the presence of various PET-LM concentrations (0–300 μg/ml) for 5 days were washed two times with PBS and further incubated in a lipolysis buffer for 1 h. The buffer was collected and glycerol content was determined in a spectrophotometric plate reader at 540 nm according to the manuals.

### Cell Cycle Progression Analysis

Cell cycle was determined by flow cytometric analysis after propidium iodide (PI) staining. In brief, the suspension (2×10^6^ cells/ml) of 3T3-L1 pre-adipocytes were divided into 6 well culture plates (2 ml/well) and incubated for 48 h in DMEM containing 10% FBS. After that, the media were switched to a serum-free DMEM and further incubated for 12 h to induce a mild synchronization. The cells were then exposed to various PET-LM concentrations (0–100 μg /ml) and cultured for additional-24 h without any treatment with FBS or adipogenic differentiating factors. Cells were harvested by a trypsinization method and resuspended in PBS containing 25% ethanol followed by incubation at −20°C for overnight. After removing the fixation solution, cells were incubated at room temperature for 30 min in a mixture containing 50 μg/ml PI and 500 units/ml RNase. The samples were stored in the dark at 4°C before cell cycle progression analysis and 10,000 cells in each experiment were counted to measure the PI intensity using a FACS Vantage® System (Becton Dickinson, San Jose, CA, USA). The cell cycle progression was determined using the ModFit LT program (Verity Software House, Topsham, ME, USA).

### DNA Synthesis Assay

DNA synthesis was determined by [methyl-^3^H] thymidine deoxyribose (TdR; Amersham Pharmacia Biotech Inc., Piscataway, NJ, USA) incorporation assay. Adipocytes were incubated in 96 multiwell culture plates in serum-free DMEM for 12 h and then treated with increasing PET-LM concentrations (0–100 μg/ml). Cells were exposed to 0.5 μCi/well TdR after 24 h of PET-LM treatment and received an additional-12 h incubation. Finally, cells were collected with a cell harvester (Inotech Biosystems International, Inc., Dietikon, Switzerland) and TdR contents incorporated into the cells were measured using a liquid scintillation counter (Packard Instrument Co.).

### Quantification of PPRAγ2, C/EBP-α, Adiponectin, Ap2, GLUT-4, Fatty Acid Synthase (FAS), and SREBP-1 mRNA Expression

The total RNA was extracted from adipocytes using RNA lipid tissue mini kit according to the manufacturer's instructions (Qiagen, Valencia, CA, USA). The extracted RNA was measured by UVS-99 micro volume UV/Vis Spectrophotometer-ACT Gene. One µg RNA was reverse transcribed using oligo (dT) and III reverse transcriptase (superscript III first stand synthesis system for RT-PCR) (Invitrogen). Real-time RT-PCR was carried out using an ABI 7500 PCR Systems (Applied Biosystems, Foster City, CA, USA). Target cDNA levels were determined by SYBR green-based real-time PCR in 20 µl reaction buffer containing 1 µl Power SYBR Green Master Mix (Applied Bio-systems), 400 µM forward (F) and reverse primers (R), and 10 ng cDNA. All PCR reactions were performed at least in triplicates, and the expression levels were normalized to the housekeeping gene β-actin in the same reaction. The primers used were follows: gtgctccagaagatgacagac (F) and ggtgggactttcctgctaa (R) for PPARγ2; gcaggaggaagatacaggaag (F) and acagactcaaatcccaaca (R) for C/EBP-α; ccgttctcttcacctacgac (F) and tccccatccccatacac (R) for adiponectin; tgtgtgatgcctttgtgg (F) and tgtgtgatgccttgtgg (R) for aP2; cccacagaaggtgattgaac (F) and ggtggagatgatgacccttt (R) for GLUT-4; cccagcccataagagttaca (F) and atcgggaagtcagcacaa (R) for FAS; and gaagtggtggagagacgcttac (F) and tatcctcaaagggctggactg (R) for SREBP-1.

### Western Blot Analysis

Whole protein lysates were prepared from differentiated 3T3-L1 adipocytes in the presence and absence of PET-LM for 5 days. Equal amounts of protein extract (20 µg/sample) were separated electrophoretically by 12% SDS-PAGE, blotted onto a PVDF membrane, and then probed with the primary and secondary antibodies. Antibodies specific to PPARγ (sc-7273), C/EBPα (sc-61), and aP2 (sc-18661) were obtained from Santa Cruz Biotechnology (Santa Cruze, CA, USA). Antibody against α-tubulin was purchased from Sigma Chemical Co.

### *In Vivo* Experiment

Six-week old female ICR mice were obtained from Orient Bio Inc. (Seoul, South Korea). Mice were housed at a constant room temperature on a 12 h light/dark cycle with free access to standard laboratory diet (AIN 93G, Feed lab Co., South Korea) and water. The use of animals in this study was approved by the Laboratory Animal Center (Permit Number: CBU 2012–0039) of Chonbuk National University (Jeonju, South Korea) and all of the experiment was carried out according to the guidelines of the Animal Care and Use Committee of the University. The mice (n = 18) were randomly divided into 3 groups of 6 mice each and caged separately before administration of PET-LM. Experimental groups were orally administrated with 200 µl distilled water containing 100 and 200 mg/kg body weight PET-LM, respectively, every two times per week for 8 weeks. Control mice were supplemented without distilled water (200 µl/head) only for the same period. All animals were weighed on 1^st^, 14^th^, 28^th^, 42^nd^, and 56^th^ days of the oral supplementation.

### Statistical Analysis

Unless otherwise specified, all data are expressed as the mean ± standard error (SE) from triplicate experiments. One-way ANOVA (Scheffe test or student *t* test) was used for multiple comparisons using the Statistical Package for the Social Sciences (SPSS) program (version 16.0) (SPSS, Inc., Chicago, IL, USA). Values of p<0.05 were considered statistically significant.

## Results

### Analysis of PET-LM Using LC-MS/MS

**Figure 1:**
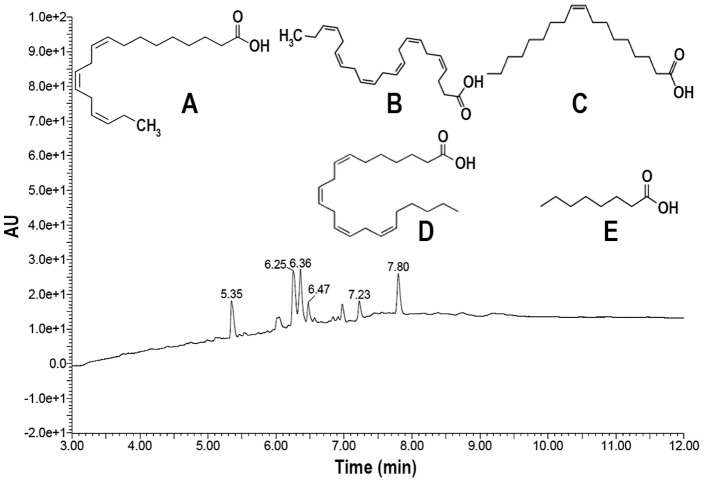
LC-MS/MS analysis of PET-LM. The result shows a LC-MS/MS profile with the chemical structures for fatty acids contained in PET-LM. A, α-Linolenic acid; B,Docosahexaenoic acid; C, Oleic acid; D, Docosatetraenoic acid; E, Caprylic acid.

LC-MS analysis showed the presence of fatty acids as the main compounds in the PET-LM (Fig. 1). These acids were determined to be α-linolenic acid, docosahexaenoic acid, oleic acid, docosatetraenoic acid, and caprylic acid, where the retention times were 5.35, 6.25, 6.36, 7.23, and 7.80 min, respectively. The peak detected at 6.47 min was not identified.

### PET-LM Facilitates Adipocyte Differentiation and Glycerol Accumulation

**Figure 2:**
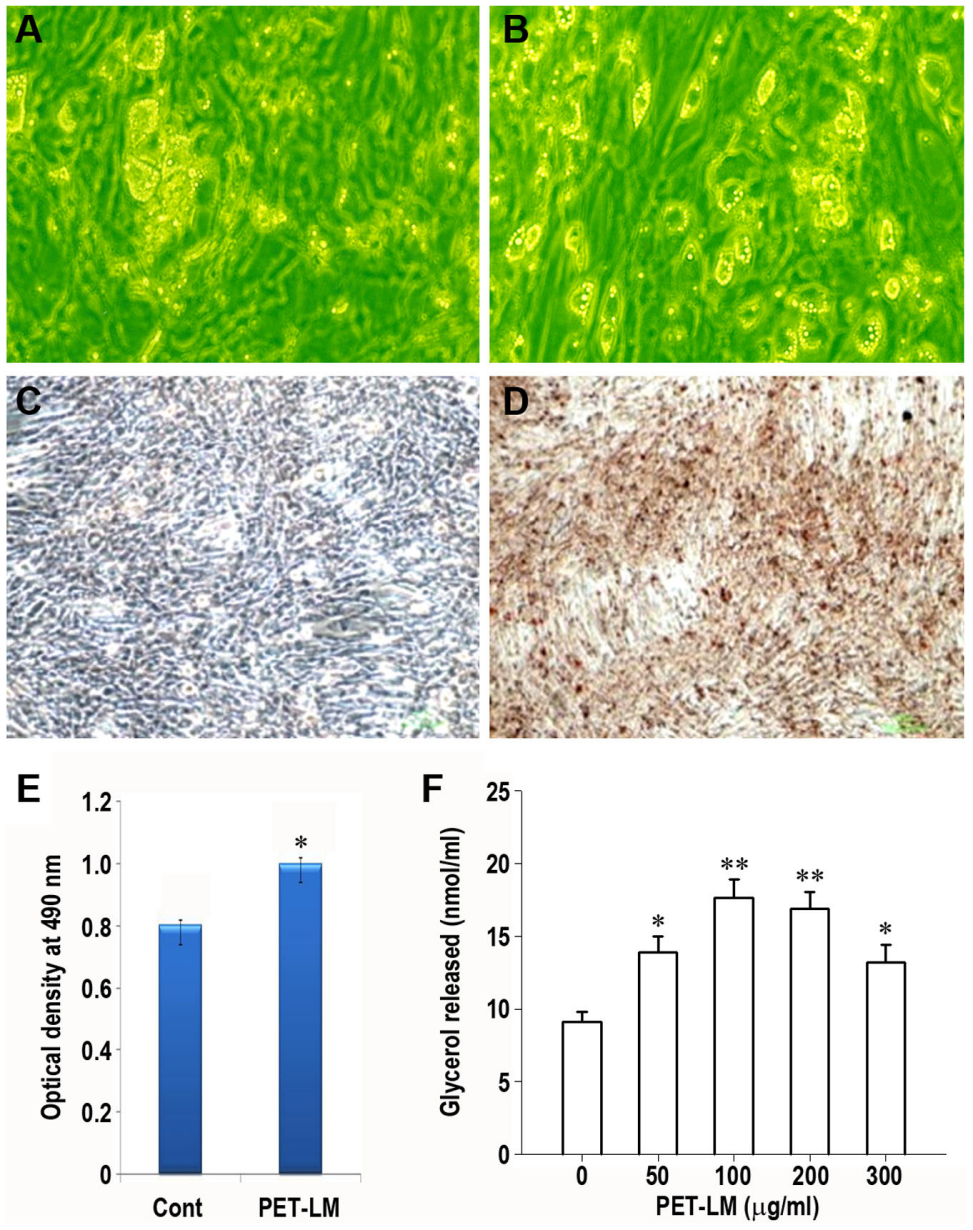
Effect of PET-LM on differentiation and lipid droplet and glycerol accumulation in 3T3-L1 adipocytes. Cells were incubated in adipogenic differentiation medium without (A and C) and with 100 µg /ml PET-LM (B and D) for 10 days. Panels A and B show a representative picture (20× magnification) from three independent experiments. The cells in panels C and D were processed for Oil Red O staining and a representative photograph (10× magnification) from triplicate experiments is shown. (E) The absorbance of Oil Red O incorporated into the differentiated cells in the presence and absence of 100 µg/ml PET-LM for 10 days was determined at 490 nm. (F) The cells were also processed for glycerol accumulation assay after 5 days of differentiation. The results show the mean ± SE from triplicate experiments. ^*^p<0.05 and ^**^p<0.01 vs. the non-treated control values.

Optic microscopic observation revealed that a few of 3T3-L1 cells were differentiated into mature adipocytes at 10 days after differentiation induction (Fig. 2A). This differentiation was accelerated by 100 µg/ml PET-LM treatment (Fig. 2B). The PET-LM-mediated facilitation of adipogenic differentiation was also detected at 5 days after differentiation (data not shown). The results from Oil Red O staining showed that the intracellular lipid accumulation was greater in the cells treated with 100 µg/ml PET-LM than the control (Figs. 2C and D). The absorbance of the dye was also higher in PET-LM (100 µg/ml)-treated cells as compared with control cells (Fig. 2E). Significant increase in lipid accumulation by PET-LM was also shown when the extract was added at 50 µg/ml (data not shown). Fig. 2F shows that the accumulation of intracellular triglycerides increased markedly in the presence of PET-LM, where the extract at 100 µg/ml revealed the most efficient effect.

### PET-LM Affects Positively Cell Cycle Progression and DNA Synthesis at Relatively Low Concentration

**Figure 3:**
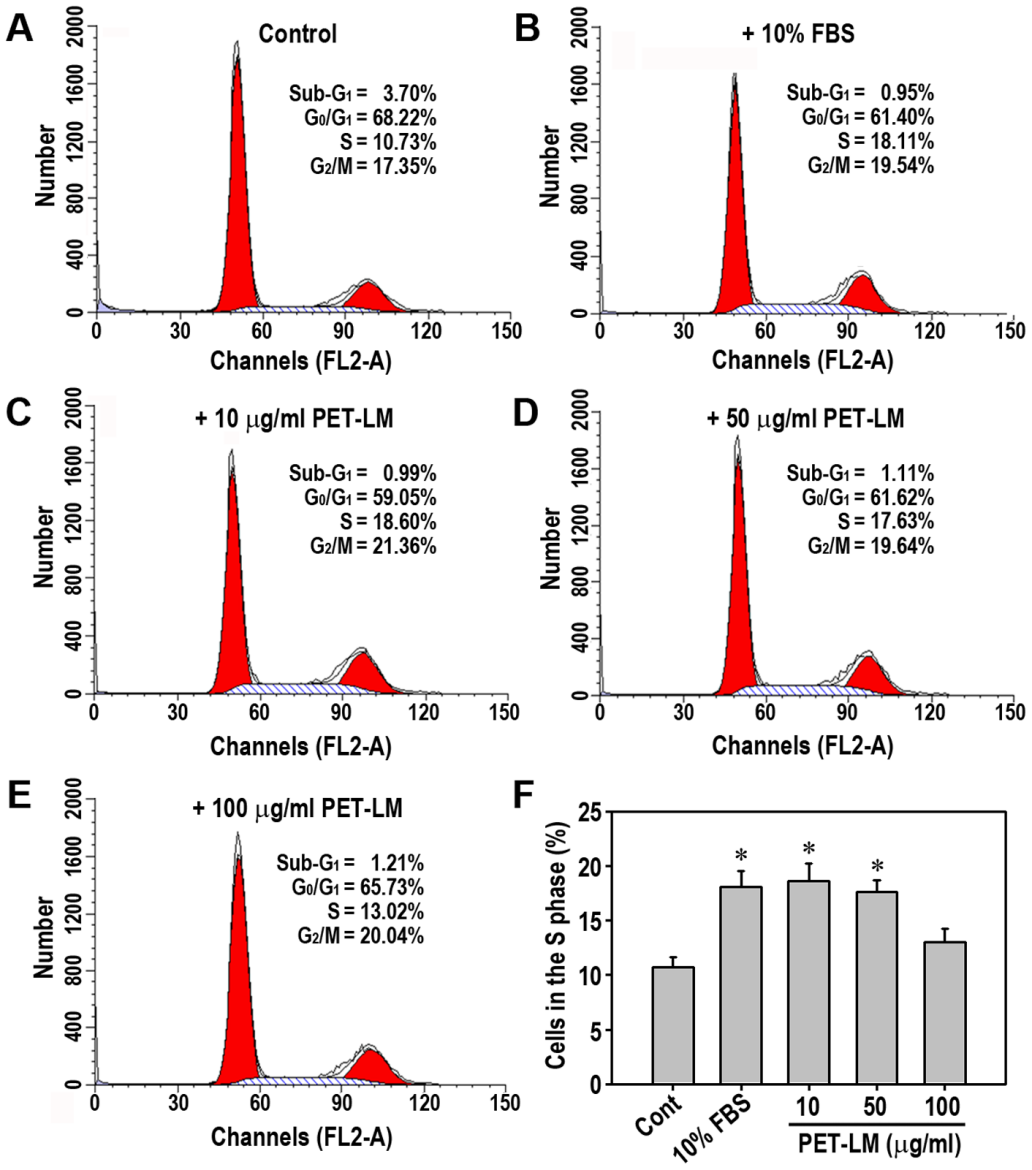
Flow cytometric analysis of cell cycle progress in PET-LM-treated 3T3-L1 pre-adipocytes. Cells were serum-starved for 12 h and then exposed to 0 (A), 10 (C), 50 (D), and 100 µg/ml PET-LM (E) for additional-24 h. The cells in panel B were incubated for the same times in the presence of 10% FBS as the positive control. Cell cycle progression was analyzed using a flow cytometer and a representative result from triplicate experiments was shown. (F) Cell population in the S phase of the cell cycle progression was calculated from triplicate experiments. ^*^p<0.05 vs. the non-treated control cells.

We subsequently analyzed cell cycle progression in PET-LM-exposed 3T3-L1 cells using flow cytometer after PI staining. Serum-deprivation of the cells led to approximate 70% synchronization in the G_0_/G_1_ phase of the cell cycle progression (Fig. 3A). Serum starvation also induced apparently the migration of cell population into the sub-G_0_/G_1_ phase with the concomitant decrease of cells in the S phase as compared with the positive control containing 10% FBS (Fig. 3B). PET-LM treatment restored the serum starvation-mediated changes in the cell cycle progression to the levels of positive controls, such that approximately 11% of the S phase and 17% of the G_2_/M phase in serum-starved cells increased to 19 and 21%, respectively, in the presence of 10 µg/ml PET-LM (Fig. 3C). These increases were not proportional to the concentration of PET-LM added (Figs. 3D and E). Fig. 3F shows significant differences in the S phase among the control and experiments, where cell population in the S phase in the groups treated with 10 or 50 µg/ml PET-LM was similar to that of the positive group containing 10% FBS.

**Figure 4:**
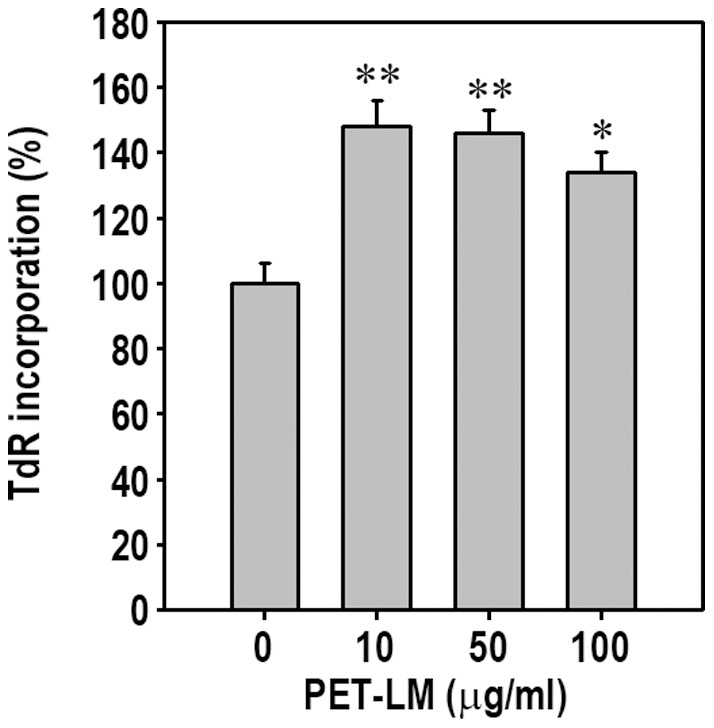
Effect of PET-LM on DNA synthesis of 3T3-L1 pre-adipocytes. Cells cultured in 96 multiwell culture plates were incubated for 24 in the presence of the indicated concentrations of PET-LM without FBS and then proliferation rates were analyzed using ^3^H-TdR incorporation. ^*^p<0.05 and ^**^p<0.01 vs. the non-treated control.

To verify whether or not PET-LM stimulates proliferation of 3T3-L1 pre-adipocytes, we performed TdR uptake assay. PET-LM treatment inhibited significantly the serum starvation-mediated decrease in TdR incorporation by the cells (Fig. 4). Inconsistent with the result from cell cycle progression analysis, the TdR level incorporated was also higher in the cells treated with PET-LM than the non-treated cells. However, PET-LM addition did not increase DNA synthesis by the cells incubated in the adipogenic differentiation medium in a significant level (data not shown). These results suggest that PET-LM partially stimulates survival in serum-starved pre-adipocytes and increases proliferation of the cells.

### PET-LM Stimulates Adipogenic Regulatory Factors at mRNA and Protein Levels During Differentiation

**Figure 5:**
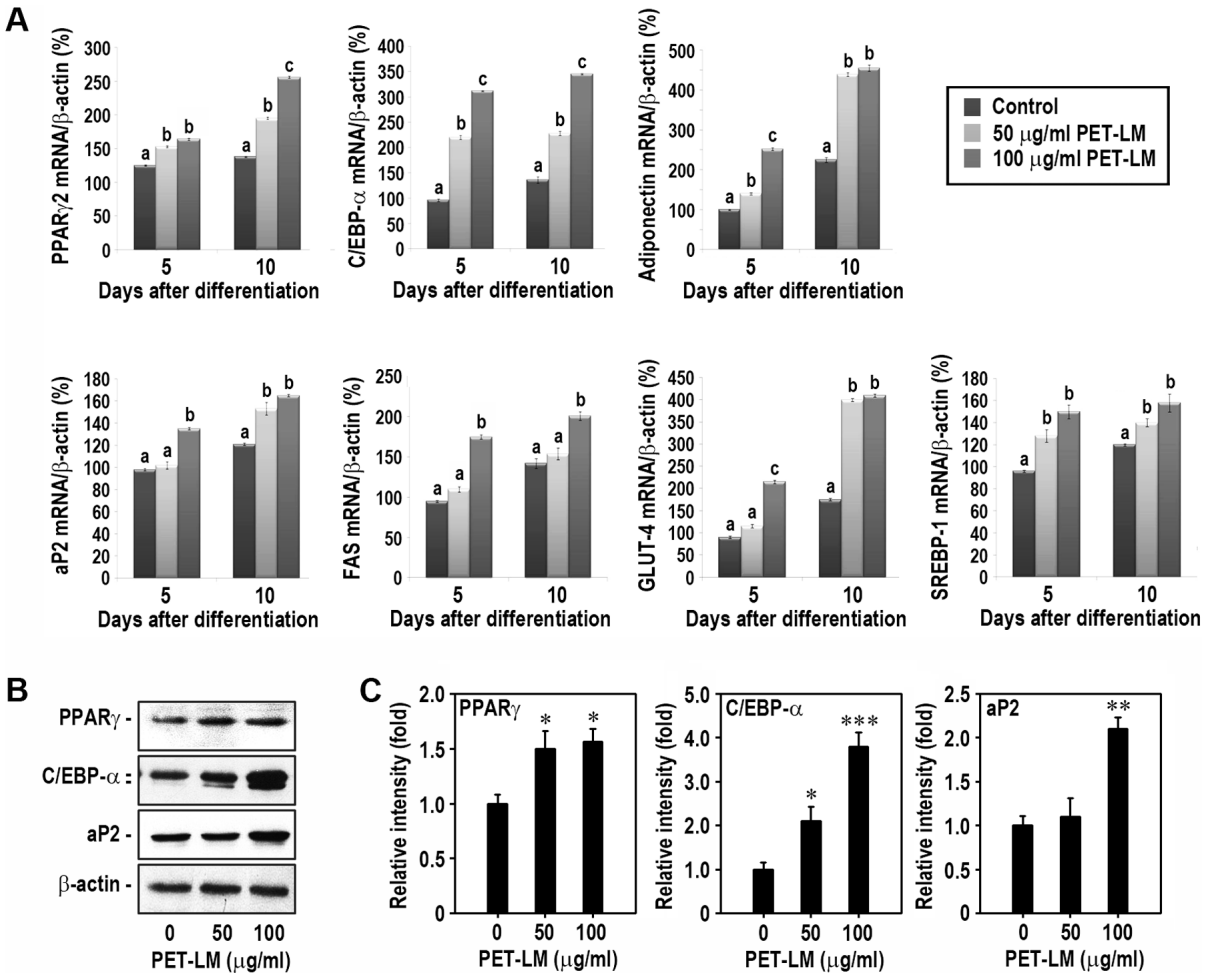
Effect of PET-LM on adipogenesis-related factor mRNA and protein expression. (A) Cells were incubated in adipogenic differentiation medium with and without 50 or 100 µg /ml PET-LM for 5 or 10 days. mRNA expression patterns of PPARγ2, C/EBP-α, adiponectin, aP2, FAS, GLUT-4, and SREBP-1 were analyzed by real-time RT-PCR. The results represent the mean ± SE of triplicate experiments. Different letters within each treatment represents significant differences among the experiments at the same incubation time (p<0.05 level). (B) In addition, differentiated 3T3-L1 cells for 5 days were processed for immunoblot analysis using antibodies specific to PPARγ, C/EBP-α, and aP2. (C) The relative fold increases in the protein induction were calculated using a densitometer after normalizing the band intensity to that of β-actin. ^*^p<0.05, ^**^p<0.01, and ^***^p<0.001 vs. the non-treated controls.

We investigated the effect of PET-LM on mRNA expression of adipogenesis-related transcriptional and co-factors by real-time RT-PCR. As shown in Fig. 5A, PET-LM treatment increased dramatically PPARγ2, C/EBP-α, adiponectin, aP2, FAS, GLUT-4, and SREBP-1 mRNA expression. PET-LM-induced mRNA increases of C/EBP-α, adiponectin, and GLUT-4 were further higher than that of other factors. The adipogenic regulatory factor mRNA levels were also greater in the cells supplemented with 100 µg/ml PET-LM, compared with the cells treated with 50 µg/ml. Comparing the mRNA expression patterns according to the incubation times, the mRNA expression levels of PPARγ2, adiponectin, ap2, FAS, and GLUT-4, but not of C/EBP-α and SREBP-1, were further higher at 10 days after differentiation than the factors were analyzed at 5 days. These results suggest that PET-LM stimulates adipogenic factor mRNA expression in a dose- and time-dependent manner. PET-LM addition also increased the protein induction of adiopogenic transcription factors in the cells (Fig. 5B). When the cells were treated with 100 µg/ml PET-LM, PPARγ, C/EBP-α, and aP2 protein levels increased approximately 1.6-, 3.8-, and 2.1-fold, respectively, relative to control cells after 5 days of differentiation (Fig. 5C).

### PET-LM Oral Supplementation Increases Body Weight of Animals

**Figure 6:**
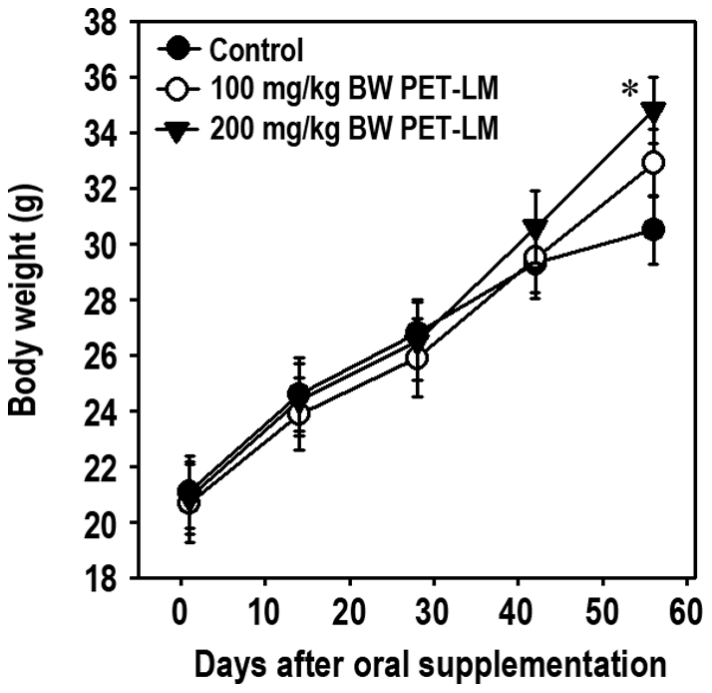
Effect of oral PET-LM supplementation on the body weight of animals. Mice (n = 6 per each experiment) were orally administered with 200 µl distilled water containing 0, 100, and 200 mg/kg body weight PET- LM every two times per week for 8 weeks. Body weight of animals was measured at various times after oral supplementation. The results represent the mean ± SE of duplicate experiments. ^*^p<0.05 indicates a significant difference between experiments at the same day.

Mice showed a gradual increase of body weight according to the experimental periods regardless of oral PET-LM feeding (Fig. 6). Comparing the body weight gained after PET-LT supplementation, mice administered with 200 mg/kg body weight of the extract showed a significant increase of body weight only after 8 weeks of supplementation as compared to control group.

## Discussion

In this study, we demonstrate that PET-LM has a potential to stimulate adipogenesis. This was evidenced by the increases in lipid droplet and glycerol accumulation, as well as in body weight after PET-LM treatment. These increases appeared to be in part dependent on PET-LM concentration added. Our present findings are consistent with a previous report showing that protocatechuic acid contained in the *aplpinia oxyphylla* enhances adipogenesis in stromal cells [13]. PET-LM treatment at relatively low concentrations (10 µg/ml) also restored completely the serum starvation-mediated reduction in the S phase of the cell cycle progression up to that of the positive control cells supplemented with 10% FBS. In addition, PET-LM addition (from 10 to 100 µg/ml) increased proliferation of 3T3-L1 pre-adipocytes incubated in a serum-free DMEM. However, there were no dose-dependent increases in the DNA synthesis and the cell cycle progression after PET-LM treatment, in that 10 µg/ml PET-LM addition showed the most efficient effect in both the assays. In this regard, we postulate that PET-LM stimulates adipogenic differentiation more predominantly than it affects proliferation or cell cycle progression of pre-adipocytes. This could be also supported by the observation that PET-LM did not affect proliferation of the cells under adipogenic differentiation (data not shown).

The mRNA expression of adipogenesis-related transcriptional factors, such as C/EBP-α and PPRAγ2, increased in PET-LM-treated 3T3-L1 cells on both 5^th^ and 10^th^ day after treatment, compared with the control cells. C/EBP-α and PPRAγ2 are the key factors to regulate adipogenic gene expression during the proliferation and differentiation periods [14], [15]. It was reported that these factors co-regulated each other gene expression [16]. C/EBP-α plays an important role for the activation and maintenance of PPRAγ2 expression during adipogenic differentiation. C/EBP-α also induces expression of genes that are involved in insulin sensitivity, lipogenesis, and lipolysis, as well as other encoding gene [17], [18]. Our present data suggest that the increased PPRAγ2 expression may continuously stimulate adipogenic differentiation in PET-LM-exposed adipocytes by directly acting on C/EBP-α. Similarly, a previous report showed that a mulberry leaf extract stimulated strongly adipogenesis in 3T3-L1 cells by enhancing PPRAγ2 and C/EBP-α [19].

Adiponectin, referred to as Adipo-Q and Acrp30, is a protein hormone secreted from adipose tissue into the bloodstream. It regulates a number of metabolic processes either by enhancing insulin sensitivity in muscle and liver or by stimulating fatty acid oxidation in many tissues [20]–[22]. Based on these roles of the adiponectin, the increase in adiponectin mRNA expression might respect a correlated lipid accumulation in adipocytes. Our present findings support such suggestion, in that PET-LM not only increases lipid droplet and glycerol accumulation in adipocytes, but also sensitively augments adiponectin mRNA expression during differentiation. This result is in parallel with a recent report that the increased lipid accumulation by a natural compound is correlated with the adiponectin level during adipocyte differentiation [19].

aP2, called fatty acid binding protein 4, is carrier protein for fatty acids and involved in adipocyte differentiation [23]. Thus, as like other adipogenic factors, aP2 mRNA expression promotes positively fatty acid metabolism and glucose uptake in cells [24]. In addition to PPARγ2, C/EBP-α, and adiponectin, PET-LM treatment triggered aP2 mRNA expression in adipocytes. PET-LM also showed a dose-dependent increase of adipogenic regulatory factors at mRNA and protein levels. This result was in part consistent with the finding that a few of natural compounds, such as epigallocatechin gallate, resveratrol, lycopene, and β-carotene, increased adipogenic factor expression at lower concentrations, whereas they decreased the expression at relatively higher concentrations [24]. We found that PET-LM at 100 µg/ml is the most efficient concentration to enhance the lipid accumulation and adipogenic factor induction at mRNA and protein levels. Indeed, no adipogegenic factor mRNA expression induced by 100 µg/ml PET-LM was additionally augmented, even at 200 µg/ml PET-LM addition (data not shown). Although we could not explain the exact reason, it is considered that at relatively higher concentrations than 100 µg/ml, PET-LM suppresses cell cycle progression and proliferation in adipocytes and eventually induces a cytotoxic effect, regardless of a culture condition, namely in growth or differentiation medium.

Fatty acid synthase (FAS) is one of the key complex enzymes involved in the lipogenesis pathway [25]. GLUT-4 plays crucial roles in the energetic and metabolic activity in adipocytes [26]. PPARγ2 and C/EBP-α coordinate the expression of genes that are involved in creating and maintaining the adipocytes phenotype, including insulin responsive GLUT, stearoyl CoA deasturase-2, and aP2 [27]. These transcription factors also stimulate the glucose uptake and insulin sensitivity pathway by up-regulating many factors that are involved in GLUT-4 signaling cascade [28]. SREBP-1 stimulates the genes that are participated in the lipogenesis by fatty acid synthase, acetyl carboxylase, glycerol-3- phosphate acyltransferase, and the lipoprotein lipase [29]–[31]. The SREBP-1 regulates the fatty acid metabolism through PPARγ2 activation [32] and also stimulates FAS and S14 gene promoters [33], [34]. According to real-time RT-PCR results, the adipogenic factors, such as FAS, GLUT-4, and SREBP-1, is also closely associated with PET-LM-induced lipid accumulation in adipocytes. Taken together, we suggest that PET-LM stimulates adipogenesis by activating adipogenesis-specific genes, where the two transcription factors, PPARγ2 and C/EBP-α, play key regulatory roles.

High fat intake is associated with fat mass development via fatty acid activation of PPARγ. Fatty acids, such as saturated, monosaturated and poly-unsaturated fatty acids, and arachidonic acid, are known to promote differentiation of clonal adipocytes [35]. Fatty acids stimulate the porcine adipocyte differentiation and their specific transcriptional and co-factor gene expression [36]. Docosahexaenoic acid, an essential n-3 poly unsaturated fatty acid, acts as a ligand of PPARγ and enhances the expression of aP2 and adiponectin by increasing PPARγ in myoblasts [37]. Triglycerides mixture, caprylic acid, and very low, low, and high density lipoproteins stimulate the proliferation and differentiation of bovine adipocytes [38]. In this study, we show that docosahexaenoic acid has the highest affinity to PPARγ2, although there is still no a direct evidence to confirm the interaction between the fatty acid and its agonist, PPARγ2. Docosahexaenoic acid has been reported to modulate differently various cellular events including proliferation and differentiation of cells according to the type of cells examined and the condition exposed. Indeed, the acid inhibited proliferation and differentiation of pre-adipocytes isolated from large yellow Croaker [39]. The docosahexaenoic acid at relative high dose (160 μM) appeared to inhibit proliferation and differentiation of rat's primary pre-adipocytes, which is in part associated with the decreased PPARγ2 mRNA expression [40]. On contrary, docosahexaenoic acid increased cellular adiponectin and its secretion at mRNA and protein levels in 3T3-L1 adipocytes, possibly by a PPARγ-involved mechanism [41]. Maternal docosahexaenoic acid increased adiponectin and normalized intrauterine growth restriction-induced changes in rat adipose deposition, thereby suggesting usefulness of the acid in treating adipose dysfunction or in improving metabolic function [42]. Considering previous reports with our present findings, it is believed that PET-LM, especially docosahexaenoic acid, regulates positively PPARγ2 gene expression and enhances other adipogenic and lipogenic gene expression, eventually stimulating adipogenic differentiation. Further, the different effects of PET-LM on proliferation and differentiation of adipocytes according to the concentrations added are considered to be associated with the increased docosahexaenoic acid levels.

## Conclusion

Adipogenesis is a very much important process regulated by several transcription factors, such as PPARγ and C/EBPs, which enhances the fat accumulation in the cells. This fat accumulation especially in the muscle (i.m. fat) is important for quality meat development in animals. The PET-LM increased slightly proliferation of serum-starved 3T3-L1 pre-adipocytes with the attendant increase in the S phase of the cell cycle progression. The extract also apparently facilitated adipogenic differentiation and lipid accumulation in the cells through the activation of various adipogenic regulatory genes. Therefore, we suggest that PET-LM stimulates adipogenesis by activating PPARγ2, which may be a plan to enhance quality meat in animals.
